# The broad-spectrum antiviral favipiravir protects guinea pigs from lethal Lassa virus infection post-disease onset

**DOI:** 10.1038/srep14775

**Published:** 2015-10-12

**Authors:** David Safronetz, Kyle Rosenke, Jonna B. Westover, Cynthia Martellaro, Atsushi Okumura, Yousuke Furuta, Joan Geisbert, Greg Saturday, Takashi Komeno, Thomas W. Geisbert, Heinz Feldmann, Brian B. Gowen

**Affiliations:** 1 Laboratory of Virology, Division of Intramural Research, Rocky Mountain Laboratories, National Institute of Allergy and Infectious Diseases, National Institutes of Health, Hamilton, Montana, United States of America; 2 Department of Animal, Diary, and Veterinary Sciences, Utah State University, Logan, Utah, United States of America; 3 Department of Microbiology, University of Washington, Seattle, Washington, United States of America; 4 Research Laboratories, Toyama Chemical Co., Ltd. Toyama, Japan; 5 Rocky Mountain Veterinary Branch, Division of Intramural Research, Rocky Mountain Laboratories, National Institute of Allergy and Infectious Diseases, National Institutes of Health, Hamilton, Montana, United States of America; 6 Galveston National Laboratory and Department of Microbiology and Immunology, University of Texas Medical Branch, Galveston, Texas, United States of America

## Abstract

With up to 500,000 infections annually, Lassa virus (LASV), the cause of Lassa fever, is one of the most prevalent etiological agents of viral hemorrhagic fever (VHF) in humans. LASV is endemic in several West African countries with sporadic cases and prolonged outbreaks observed most commonly in Sierra Leone, Liberia, Guinea and Nigeria. Additionally several cases of Lassa fever have been imported into North America, Europe and Asia making LASV a global threat to public health. Despite this, currently no approved therapeutic or vaccine exists to treat or prevent LASV infections. Here, using a passaged strain of LASV that is uniformly lethal in Hartley guinea pigs, we demonstrate that favipiravir, a broad-spectrum antiviral agent and leading treatment option for influenza, has potent activity against LASV infection. In this model, once daily treatment with favipiravir significantly reduced viral titers in tissue samples and reduced mortality rates when compared with animals receiving vehicle-only or ribavirin, the current standard of care for Lassa fever. Favipiravir remained highly effective against lethal LASV infection when treatments were initiated nine days post-infection, a time when animals were demonstrating advanced signs of disease. These results support the further preclinical evaluation of favipiravir for Lassa fever and other VHFs.

Viral hemorrhagic fevers (VHFs) are among the most deadly and feared group of diseases in humans and for most no approved vaccine or treatment exists^1^. Members of at least four families (*Bunyaviridae*, *Filoviridae*, *Arenaviridae* and *Flaviviridae*) are known to cause VHFs, though more etiological agents are likely to exist. While some VHFs are associated with sporadic but potentially explosive outbreaks, others are essentially endemic in specific geographical regions and are often associated with high annual incidence rates. With an estimated 300,000–500,000 infections annually, Lassa virus (LASV, *Arenaviridae*) is a perfect example of the latter group^2^,^3^. In humans, LASV infection can cause a wide variety of disease manifestations from essentially subclinical to severe hemorrhagic fever characterized by multi-organ failure and death, a condition known as Lassa fever (LF)^2^. The overall fatality rate for LF is between 1–2%; however, rates can increase to greater than 50% in nosocomial settings and community-based outbreaks^4^,^5^.

LASV is a prototypical member of the *Arenaviridae*; it has a bi-segmented RNA genome which produces five viral proteins using an ambi-sense coding strategy. In nature, LASV is maintained in the multi-mammate rat, *Mastomys natalensis*^6^. Although the ubiquitous *M. natalensis* is found across sub-Saharan Africa, evidence of infected rodents has exclusively been noted in Western African countries. Due to this, cases of LF are most commonly observed in Sierra Leona, Liberia, Guinea and Nigeria. These four countries represent the regions historically considered endemic for LASV/LF; though it is becoming increasingly clear that other West African nations, including Mali, Cote d’Ivoire, Benin and Ghana are also at risk for sporadic cases and potentially explosive outbreaks of LF^7^,^8^,^9^,^10^. Additionally, several imported cases of LF originating from West Africa have been diagnosed in Asia, the Americas, and most commonly Europe, making LASV/LF a global concern for human health^11^.

The high annual incidence of LF in West Africa suggests a prophylactic vaccination strategy would be the most effective way of reducing the burden of LASV infection in this population. However, the lack of approved vaccines with no candidates currently in clinical trials necessitates the evaluation of therapeutic options, preferably those already approved for human use, for treating LASV infections and LF disease. To that end ribavirin, a broad spectrum antiviral agent which is licensed for the treatment of hepatitis C, is often used off-label to treat patients diagnosed with LF. Although ribavirin therapy has been shown to reduce the morbidity and mortality associated with LF, its limited efficacy is reliant on treatment initiation within 6 days of disease onset^12^.

Favipiravir (T-705; 6-fluoro-3-hydroxy-2-pyrazinecarboxamide) is a novel antiviral agent recently approved in Japan as an anti-influenza drug and Phase 3 clinical studies have been completed in the United States for the same indication. Previous studies demonstrated that favipiravir inhibits the RNA-dependent RNA polymerase of influenza and it is effective against all strains and serotypes that it has been tested^13^,^14^,^15^,^16^,^17^. However, like ribavirin, *in vitro* and/or *in vivo* studies suggest that favipiravir exhibits broad-spectrum antiviral activity against a variety of RNA viruses, including alpha-, paramyxo-, picorna-, and caliciviruses, as well as etiological agents associated with VHFs including bunya-, flavi-, filo- and arenaviruses^15^,^17^,^18^,^19^,^20^,^21^,^22^,^23^,^24^,^25^,^26^,^27^,^28^,^29^. In most instances, *in vitro* studies have shown that the antiviral activity of favipiravir is similar to or better than that of ribavirin for most RNA virus^15^,^17^. Moreover, the safety of the compound has been thoroughly evaluated by the Japanese Ministry of Health, Labour and Welfare, and the United States Food and Drug Administration (FDA). Clinical evaluation during the recent ongoing Ebola outbreak suggests that favipiravir is well tolerated by those receiving oral treatment in West Africa Ebola treatment centers^30^. Considering the above, favipiravir should be further evaluated both in animal models as well as clinical trials for a number of RNA viral infections.

Recently, favipiravir was shown to effectively prevent lethal disease associated with Pichindé virus infection in guinea pigs and hamsters which are surrogate disease models for VHFs of arenaviral etiology^21^,^26^,^27^. To date, the effectiveness of favipiravir has not been evaluated against pathogenic Old World arenaviruses. Therefore, in the present study, we investigated the antiviral efficacy of the compound in both cell culture and *in vivo* using using a guinea pig-adapted strain of LASV-Josiah (GPA-LASV) which is uniformly lethal in Hartley outbred guinea pigs.

## Results

### *In vitro* effect of favipiravir on infectious LASV titers

**Figure 1:**
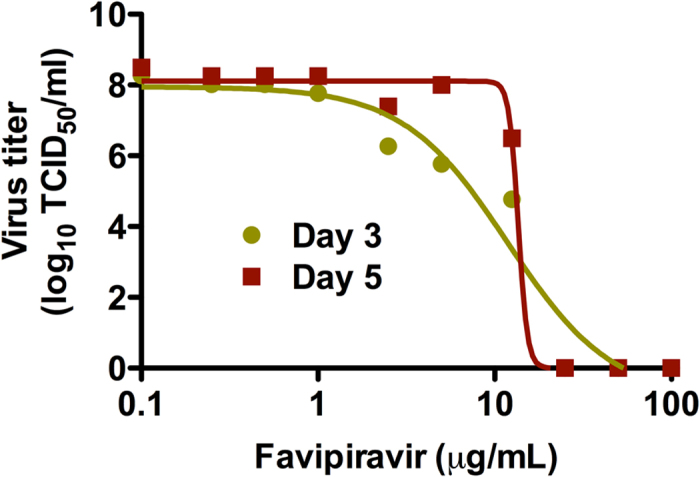
*In vitro* inhibition of LASV by favipiravir. Vero cells were infected with wild-type LASV (Josiah, m.o.i. = 0.01) and cultured in the presence of increasing concentrations of favipiravir. Linear regression analysis conducted on infectious LASV titers in supernatants collected at 3 and 5 days post-infection revealed a clear dose response with an EC_90_ of between 1.7 and 11.1 μg/mL, respectively.

Treatment of LASV infected Vero cells with favipiravir resulted in diminished infectious viral titers in a dose dependent manner (Fig. 1). Analysis of infectious LASV titers (TCID_50_) in supernatants collected at day 3 and 5 post-infection from Vero cell cultures treated with varying concentrations of favipiravir resulted in EC_90_ values of 1.7 and 11.1 μg/ml, respectively. As expected, no toxicity was observed at the highest concentration tested (100 μg/ml), translating to SI values of >59 and >9 for the day 3 and day 5 assays, respectively.

### Assessment of GPA-LASV infection in outbred guinea pigs for challenge dose determination for antiviral efficacy studies

**Figure 2:**
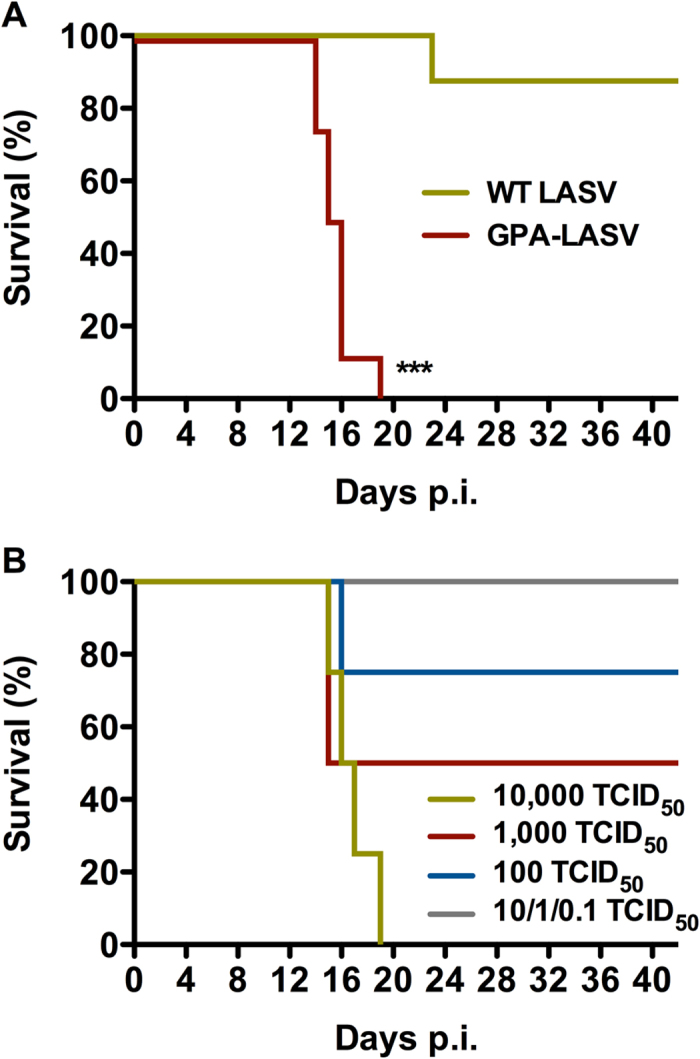
Titration of the GPA-LASV Josiah strain in outbred guinea pigs. (**A**) Comparison of survival rates following challenge with wild-type (WT) LASV and GPA-LASV. Groups of eight outbred guinea pigs were infected i.p. with 10,000 TCID_50_ and monitored for disease progression and survival. (**B**) In order to determine challenge dose, groups of four outbred guinea pigs were inoculated i.p. with ten-fold serial dilutions of GPA-LASV and monitored for disease progression and survival. The LD_50_ was estimated at 1,000 TCID_50_. ***p < 0.001 compared to WT LASV infection.

Prior to conducting the favipiravir efficacy studies we sought to ensure the uniform lethality of GPA-LASV in outbred Hartley guinea pigs and to determine an approximate LD_50_ in these animals. All eight guinea pigs infected with GPA-LASV in the first experiment developed severe disease requiring euthanasia between days 14 and 19 post-infection with an average time to lethal disease of 15 days. In contrast, only a single animal infected with wild-type LASV Josiah developed severe disease and was euthanized 23 days post-infection (Fig. 2A). In a follow-up study, groups of four animals were infected with ten-fold serial dilutions of GPA-LASV. Animals which received low doses (10^−1^ to 10^1^ TCID_50_) were refractory to lethal disease with essentially no signs of infection. A single animal infected with 10^2^, two animals infected with 10^3^, and all animals infected with 10^4^ TCID_50_ of GPA-LASV developed severe disease (Fig. 2B). The approximate LD_50_ for GPA-LASV in outbred Hartley guinea pigs was calculated to be 10^3^ TCID_50_. Based on these results subsequent experiments were conducted with a challenge dose of 10^5^ TCID_50_, which is equivalent to 100 × the LD_50_.

### *In vivo* efficacy of favipiravir treatment on lethal LASV infection in guinea pigs

**Figure 3:**
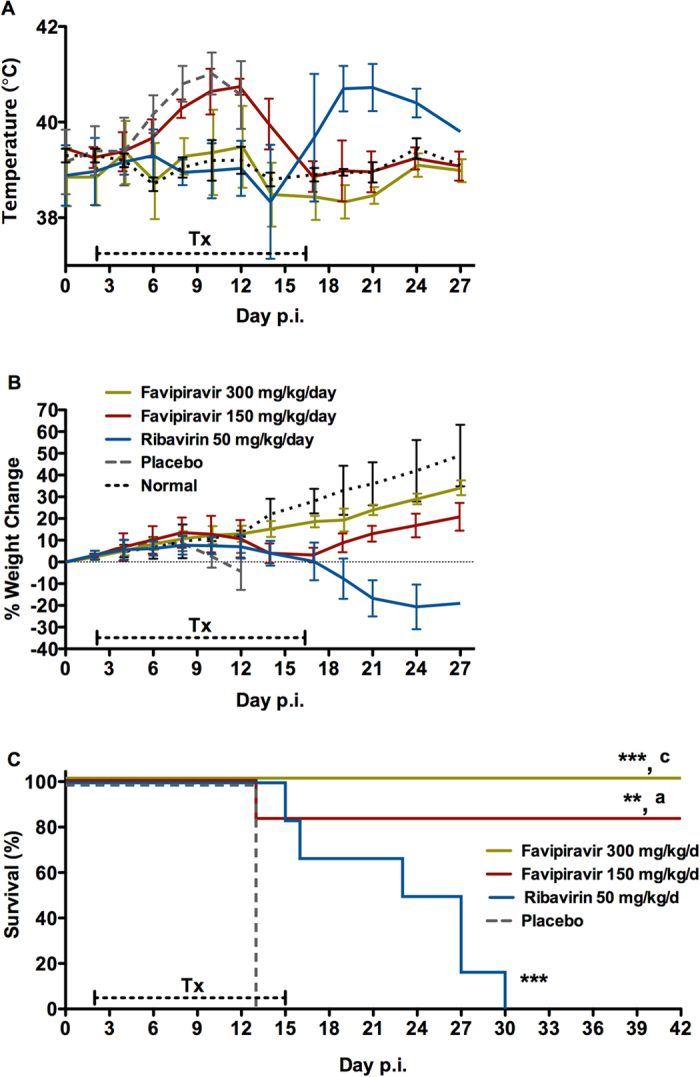
Treatment of lethal LASV infection in guinea pigs with favipiravir beginning 48 hours after challenge. Groups of nine guinea pigs were challenged with a lethal dose of GPA-LASV and treated s.c. once daily for two weeks with favipiravir (150 or 300 mg/kg/d), ribavirin (50 mg/kg/d) or vehicle placebo beginning 48 hours after challenge. At a time when control (vehicle treated) animals were demonstrating signs of advanced disease, three animals per group were euthanized for sample collection. The remaining six animals per group were monitored for (**A**) temperature, (**B**) body weight, and (**C**) survival for up to 42 days post-infection (p.i.). *p < 0.05, **p < 0.01, ***p < 0.001 compared to placebo; ^a^*p* < 0.05 and ^c^*p* < 0.001 compared to ribavirin.

Based on previous studies evaluating the efficacy of favipiravir against Pichindé virus in hamsters, a surrogate model for LF, and Junin virus, a New World arenavirus, in guinea pigs; doses of 150 and 300 mg/kg/d were selected for the initial efficacy study^21^,^26^,^31^. Since ribavirin is often used to treat cases of LF, an additional group treated with ribavirin was included for comparison. Placebo treated animals developed a fever (defined here as a body temperature of ≥40 °C) beginning on day 6 post-infection which continued until the time of euthanasia (Fig. 3A). Although weight loss was variable in these animals, ranging from 8% to >20% loss from peak weight, the entire group was euthanized between 12 and 13 days post-infection based on overall condition (Fig. 3). Animals treated daily with 150 mg/kg favipiravir also developed a fever between days 8 and 12 post-infection which coincided with weight loss between 2 and 16% of peak body weight. A single animal in this group expired on day 12 post-infection; however it was not overtly ill and its death was likely due to difficulties with recovering from the isoflurane anesthesia used to sedate the animals prior to treatments. In contrast, animals treated at 300 mg/kg/d were completely protected against GPA-LASV infection with no weight loss noted across the entire group and only two animals developed a fever, though only on days 10 and 12 post-infection. Interestingly, in the ribavirin treated comparison group, two animals succumbed to infection on the final day of treatment (day 16 post-infection) and the remaining animals in this group quickly developed fever after cessation of treatment and rapidly lost weight (5 to >20% of peak body weight). The remaining four animals in this group succumbed to infection or were euthanized within 13 days (days 23 to 29 post-infection) after treatments were completed. Mock-infected animals treated with the high dose of favipiravir did not show any adverse signs associated with daily treatments.

Compared to the control (placebo treated) animals, the survival rates in favipiravir-treated animals were statistically significant with *p* values of <0.001 and <0.01 for the 300 and 150 mg/kg/d treatment groups, respectively (Fig. 3C). Although the ribavirin treated group demonstrated a significant delay in time to lethal disease compared to the control group with a *p* value of 0.001, survival outcome in favipiravir-treated animals was significantly better compared to the ribavirin group with *p* values of <0.001 and <0.05 for the 300 and 150 mg/kg/d treatment groups, respectively.

**Figure 4:**
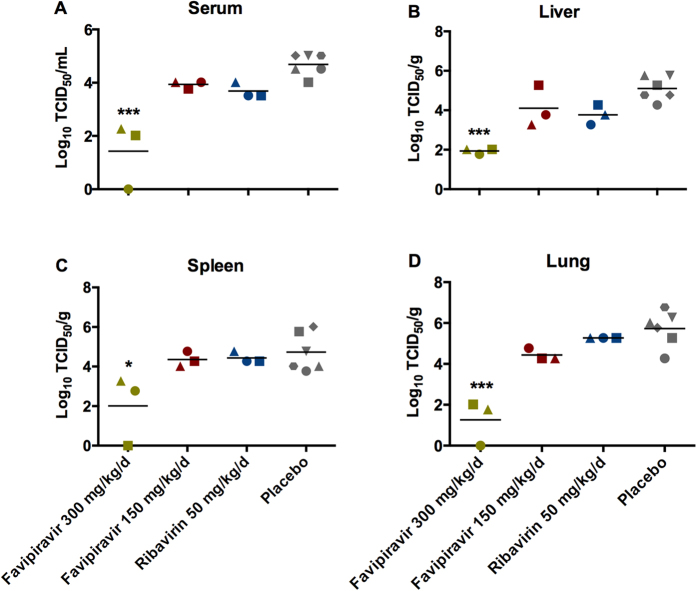
Effect of favipiravir treatment on viral loads in guinea pigs. In the first *in vivo* efficacy study, three guinea pigs per treatment group (150 or 300 mg favipiravir/kg/d, 50 mg ribavirin/kg/d, or placebo) were euthanized for sample collection on day 12 post-infection, a time when control animals were displaying signs of terminal disease. Infectious titers in (**A**) Serum, (**B**) Liver, (**C**) Spleen and (**D**) Lung are shown. *p < 0.05, ***p < 0.001.

**Figure 5:**
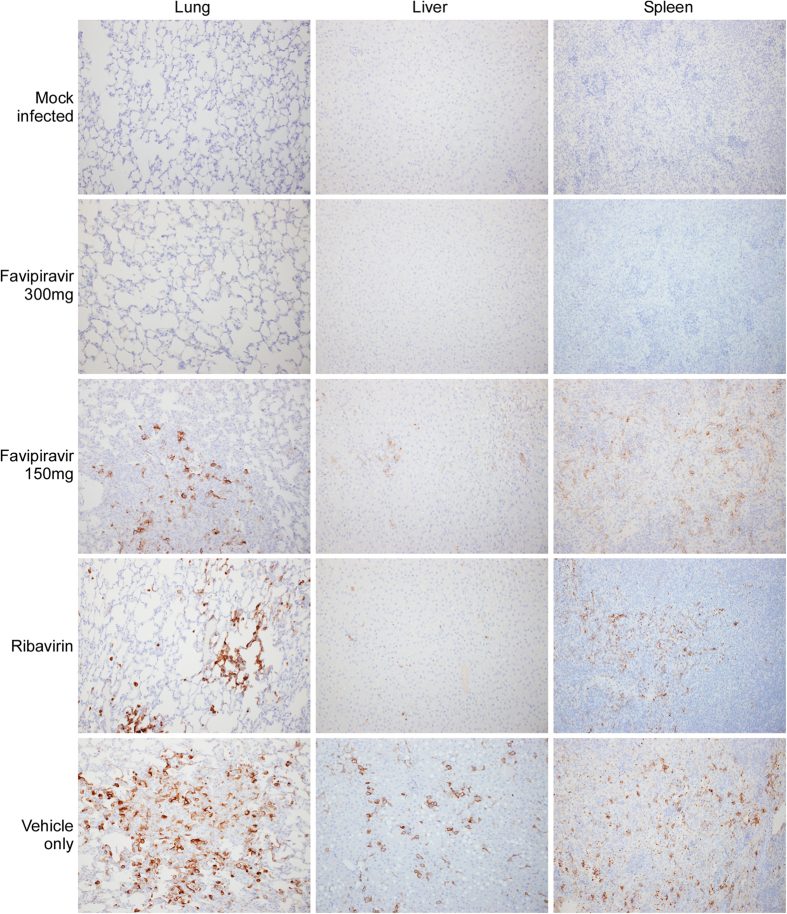
Immunohistochemical analysis of tissues from animals infected with GPA-LASV and treated daily with favipiravir, ribavirin or placebo. Tissue samples from infected guinea pigs treated daily with favipiravir (150 or 300 mg/kg/d), ribavirin (50 mg/kg/d) or placebo were collected at 12 days post-infection and examined for the presence of LASV antigen (glycoprotein, GP) using a GP-specific monoclonal antibody according to standard immunohistochemistry techniques.

Viral titrations conducted on serum and tissue samples from each group of animals provide corroborating support for the efficacy of favipiravir against LASV infection (Fig. 4). Infectious viral titers in samples collected from animals treated daily with 300 mg/kg favipiravir were reduced by 2–3 logs compared with the placebo control group. Viral titers in tissues from the 150 mg/kg favipiravir treatment group were similar to those from the ribavirin comparison group and were approximately 1 log lower than the placebo control groups. Consistent with these findings, LASV antigen was not detected by IHC analysis in lung, liver or spleen samples collected from animals treated daily with 300 mg/kg favipiravir (Fig. 5). LASV antigen was detectable in all tissues examined from both the low dose favipiravir group as well as the ribavirin comparison group; however, the pattern of antigen staining in these tissues was less intense and more focal compared to the diffuse and coalescing patterns noted in the placebo controls (Fig. 5).

**Figure 6:**
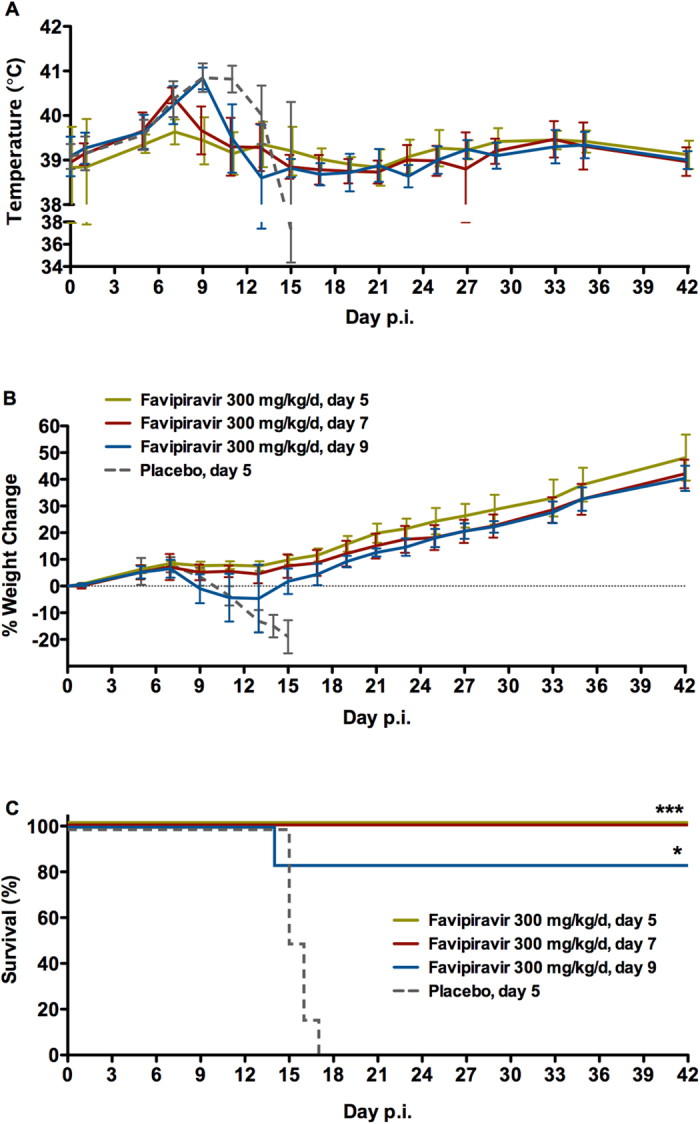
Favipiravir treatment of advanced LASV infection in guinea pigs. Groups of six guinea pigs were challenged with a lethal dose of GPA-LASV. Beginning on days 5, 7, and 9 post-challenge, favipiravir treatment (300 mg/kg/d, once daily s.c. for 14 consecutive days) was initiated in the respective group of animals. A control, vehicle placebo treatment group was included with treatments commencing at 5 days post-infection and (**A**) temperature, (**B**) body weight, and (**C**) survival were monitored for up to 42 days post-infection (p.i.). *p < 0.05, ***p < 0.001.

In the second efficacy experiment, we sought to determine if delayed treatment with favipiravir would remain effective in preventing lethal LASV infection. To that end, four groups of six guinea pigs were infected with GPA-LASV as in the first experiment and treatments for three groups with 300 mg/kg/d of favipiravir were initiated at 5, 7, and 9 days post-infection, while the forth control group received vehicle placebo beginning on day 5 post-infection. Once daily treatments for each group lasted for 14 consecutive days. The six control, placebo treated animals developed severe disease indicated by fever beginning on day 7 and significant weight loss (>20%) which first began on day 9 post-infection (Fig. 6A,B). The control animals succumbed to infection or were humanly euthanized between days 14 and 16 post-infection (Fig. 6C). Fever was uniformly noted across all treatment groups beginning on day 7 post-infection with the exception of the group which first received favipiravir on day 5. In this group, only a single animal had a temperature exceeding 40 °C and only on day 7 post-infection. Similarly, none of the animals in this group experienced any significant decreases in body weight. All six animals which began treatments on day 7 post-infection were febrile at time of treatment onset; however, fever resolved in four of these animals before day 9 post-infection (1–2 days post-treatment onset). Body temperature in the remaining two animals in this group returned to normal on or before days 11 and 15 post-infection and similar to the day 5 treatment group, no animals in this group experienced any significant decreases in body weight. Despite having increased temperatures for 2–4 days, the fever observed in animals in the day 9 treatment group resolved within 2–4 days after treatment onset. One animal in this group rapidly lost weight (>20% of peak body weight) and quickly became hypothermic (temperature <36 °C) resulting in it being humanely euthanized. The remaining animals in this group quickly recovered and only lost between 5 and 10% of peak body weight. The survival rates in all three treatment groups are statistically significant with *p*-values of <0.01 for the day 5 and day 7 treatment onset groups and <0.05 for the day 9 treatment onset group.

All surviving animals from both efficacy experiments demonstrated high titer (≥6400) anti-LASV nucleocapsid IgG antibodies in convalescent (day 42) serum samples; thereby confirming these animals were exposed to LASV.

## Discussion

With an annual incidence rate of up to 500,000 infections, LASV is easily the second most prominent etiological agent of VHF, behind only dengue virus. In addition to a large endemic region comprising many countries in West Africa, dozens of imported cases of LF have been diagnosed in North America, Asia and Europe, which makes LASV/LF a global public health concern. Despite this, LF remains one of the world’s most neglected diseases with no licensed therapeutic or vaccine options currently available.

A limitation to evaluating potential therapeutics against LASV infection or LF disease is lack of readily available rodent models which can be utilized in initial efficacy studies. Currently, the only immuno-competent small animal disease models for the study of LASV infection are strain 13 and less commonly strain 2 inbred guinea pigs^32^. Although these models are based on infection with wild-type LASV isolates, a lack of commercial vendors to purchase these animals from limits their availability. Even institutes with in-house breeding colonies are hampered for the types of studies described here in that it can be difficult to produce sufficient numbers of guinea pigs within a limited size/age range at one time. Despite the commercial availability of outbred Hartley guinea pigs their use as an infection or disease model for LASV/LF is hampered by lethality rates of approximately 30%^33^, meaning antiviral studies like the one described here would require considerably larger group sizes in order to determine statistically significant differences between treatment and control animals.

In order to overcome these hurdles we utilized a strain of LASV Josiah which had been passaged from terminally-ill outbred guinea pigs into healthy animals, four times. The resultant isolate (GPA-LASV, unpublished data) demonstrated a uniformly lethal phenotype at challenge doses of 10^4^ TCID_50_ or greater (LD_50_ = 10^3^ TCID_50_). Histological abnormalities in this model recapitulated those commonly observed in the inbred strain 13 model^34^ with classical LASV lesions noted in liver (hepatocellular degeneration and lymphohistiocytic hepatitis), spleen (sinus histiocytosis) and lungs (interstitial pneumonia) of these animals (unpublished data). Combined, the GPA-LASV Hartley guinea pig model provided us a convenient small animal model to evaluate the antiviral efficacy of favipiravir against LASV infection and ensuing disease.

Following infection with 100 × the LD_50_ of GPA-LASV, placebo treated guinea pigs began to show signs of disease, including increased temperature and weight loss on days 6 and 8, respectively. Other signs of disease, including lethargy, ruffled fur, recumbency and reddening of ears and footpads appeared between days 9 and 11 post-infection. Animals in the placebo treatment group succumbed to infection or were euthanized between days 12 and 16 post-infection. Since ribavirin is currently the standard of care for LF patients, a comparison group was included in these studies. Animals treated with ribavirin appeared relatively normal throughout the treatment phase, although animals essentially only maintained their body weight with very minimal gains noted. Within two days of the end of treatments the four remaining animals in the ribavirin treatment group developed signs of disease and although treatment provided a prolonged time to death, it did not improve the overall survival rate. Similar results with ribavirin treatment (albeit at half the dose utilized here and by i.p. injection) in a guinea pig model of LF have been previously noted^35^. In contrast, treatment with the low and high doses of favipiravir provided significantly increased survival rates with only a single animal in the low dose group requiring euthanasia; though primarily because of difficulties recovering from anesthetic as opposed to disease severity. Nevertheless, animals in the 150 mg/kg/d treatment group experienced mild to moderate signs of disease which were delayed by 1–2 days compared to the control group, all of which resolved within 7–10 days of onset. Despite demonstrating similar infectious titers and IHC viral staining patterns to the ribavirin treatment group, animals in the low dose favipiravir group did not experience a recrudescent LASV infection after treatments were stopped.

Similar to recent favipiravir efficacy studies against Pichindé virus infection in guinea pigs^21^, animals receiving 300 mg/kg/d were completely protected from lethal LASV infection with no signs of disease observed throughout the course of the initial study. Supporting this, tissue samples collected at 12 days post-infection revealed infectious viral titers as much as 3 logs lower than the control animals with no viral antigen detectable by IHC. Importantly, treatment with 300 mg favipiravir/kg/d remained protective even when treatments were initiated several days after clinical signs of disease, a scenario which more closely mimics what would be expected for human cases of LF. Although animals which began treatment at 1 week or greater post-infection all experienced signs of disease, their condition quickly improved within a few days of the initiation of treatment. Only a single animal (treatment initiation at day 9 post-infection) did not respond fully to favirpiravir therapy with no resolution of signs of disease and eventual death. To put the concentration of favipiravir utilized in these studies in the context of human dosing, based on body surface area conversions^36^, 300 mg/kg in a guinea pig represents a human dose of 65 mg/kg, which is less than the 100 mg/kg day 1 loading dose (6 g of favipiravir, assuming 60 kg person; reduced to 40 mg/kg/d for days 2–10) being evaluated in the ongoing Ebola outbreak^30^.

Due to the often non-descript prodrome associated with many etiological agents of VHFs, the development and characterization of broad spectrum antivirals is vital to increase life expectance as well as to curtail potential human-to-human spread of some of these agents. Our present findings suggest that in addition to other emerging VHF agents including Ebola, Crimean-Congo-hemorrhagic fever, Andes and Junin viruses, favipiravir is highly effective at preventing lethal disease associated with LASV infection, even when treatments are initiated several days after the onset of clinical disease. Combined with its excellent tolerability in humans, these studies further suggest that favipiravir should be a front line treatment option for patients demonstrating symptoms of VHF.

## Methods

### Ethics statement

*In vivo* efficacy studies were approved by the Institutional Animal Care and Use Committee of the Rocky Mountain Laboratories (RML). Animal work was conducted adhering to the institution’s guidelines for animal use, and followed the guidelines and basic principles in the United States Public Health Service Policy on Humane Care and Use of Laboratory Animals, and the Guide for the Care and Use of Laboratory Animals by certified staff in an Association for Assessment and Accreditation of Laboratory Animal Care (AAALAC) International accredited facility.

### Biosafety

All work with infectious LASV and potentially infectious materials derived from animals was conducted in a Biosafety Level 4 (BSL 4) laboratory in the Integrated Research Facility of the Rocky Mountain Laboratories (RML), National Institute of Allergy and Infectious Diseases (NIAID), National Institutes of Health (NIH). Sample inactivation and removal was performed according to standard operating protocols approved by the local Institutional Biosafety Committee.

### Animals

Outbred male Hartley guinea pigs (300–350 g) (Charles River, Wilmington MA) were ear-tagged for identification and group housed, 2 per box in an isolator caging unit. For the *in vivo* efficacy studies animals were also subcutaneously implanted with IPTT-300 electronic transponders for temperature measurement with a DAS 6002 hand-held scanner (BMDS, Seaford, DE). Animals were acclimated to BSL 4 environment for one week prior to challenge and, in the antiviral studies, sorted so that the average body weights of each treatment group was similar.

### Virus

*In vitro* studies were conducted with LASV strain Josiah^37^. *In vivo* experiments utilized a LASV strain Josiah that was passaged four times in Hartley guinea pigs (guinea pig-adapted LASV, GPA-LASV) resulting in a uniformly lethal phenotype in these animals (Safronetz, Feldmann, Geisbert, unpublished data). In the first *in vivo* experiment a comparison group of animals infected with wild-type LASV Josiah was included. Both viruses were propagated in Vero cell culture and titered using standard 50% tissue culture infectious dose (TCID_50_) infectivity assays and the Reed-Muench formula.

### Antiviral compounds

Favipiravir was provided by the Toyama Chemical Company, Ltd. (Toyama, Japan) and Ribavirin was purchased from R & D Systems (Minneapolis, MN). For *in vitro* studies, compounds were dissolved directly in cell culture media (see below). For *in vivo* studies, the antiviral compounds were prepared in sterile water supplemented with 74.6 mg/ml meglumine excipient.

### *In vitro* antiviral efficacy studies

In order to determine the 90% effective concentration (EC_90_) of favipiravir against LASV, nearly confluent (>95%) monolayers of Vero cells were infected with LASV Josiah at a multiplicity of infection (m.o.i.) of 0.01. After a 1 hour absorption, cells were washed and the inoculum replaced with culture media (Dulbecco’s modified Eagle’s medium supplemented with 2% fetal bovine serum, 100 U/ml penicillin, 100 μg/ml streptomycin and 2mM _L_-glutamine) containing varying concentrations (0, 0.1, 0.25, 0.5, 1, 2.5, 5, 12.5, 25, 50 or 100 μg/ml) of favipiravir. On days 3 and 5 post-infection supernatants were collected and infectious viral titers were determined as above. Cell viability was assessed visually at the time of sample collection. The EC_90_, which indicates the dose at which the viral titer is reduced by 1 log_10_, was calculated using basic linear regression analysis on the points in the linear range (r-squared >0.9). The maximum concentration of favipiravir utilized in these *in vitro* studies (100 μg/ml) is well below the documented 50% cytotoxic concentration (CC_50_) in Vero cells (>500 μg/ml)^38^. The selectivity index (SI) was calculated as CC_50_/EC_90_.

### Determination of challenge dose for the GPA-LASV

In order to determine an appropriate challenge dose with the GPA-LASV we first conducted a pilot experiment to ensure uniform lethality of GPA-LASV in outbred Hartley guinea pigs. To that end two groups of eight guinea pigs were infected with 1 × 10^4^ TCID_50_ of LASV or GPA-LASV (both strain Josiah) via intraperitoneal (i.p.) injection. Animals were monitored for disease progression for 42 days and were only euthanized if they reached a pre-determined clinical score indicative of advanced terminal disease (based on weight loss, mobility and respiration). In a second experiment, six groups of four guinea pigs were challenged with ten-fold serial dilutions of GPA-LASV (dose range 1 × 10^4^ to 1 × 10^−1^ TCID_50_) by i.p. injection. Animals were monitored for disease progression for 42 days and were euthanized when they demonstrated signs of advanced terminal disease.

### *In vivo* antiviral efficacy studies

The *in vivo* efficacy studies were conducted in two independent experiments. In the first study, three groups of nine guinea pigs were inoculated with 1 × 10^5^ TCID_50_ GPA-LASV representing 100 × the 50% lethal dose (LD_50_) via i.p. injection. Beginning on day 2 post-infection, animals were treated for 14 consecutive days with favipiravir (150 or 300 mg/kg/d) or ribavirin (50 mg/kg/d). Since oral gavage is not recommended for guinea pigs, treatments were administered with once daily subcutaneous (s.c.) injections. A control group of six guinea pigs was inoculated with GPA-LASV as above and treated according to the same schedule with vehicle placebo. Additionally, three guinea pigs were mock-infected and included as normal controls. Animals were subjected to mild isoflurane anesthesia prior to each treatment. Weights and body temperatures were recorded every second day. At a time when control animals were showing signs of advanced, terminal disease three animals per treatment group were euthanized and tissue samples collected to assess the extent of viral replication. The remaining six animals per group were monitored for disease progression and survival for 42 days.

In the second experiment the efficacy of delayed treatment onset with favipiravir was assessed. Three groups of six guinea pigs were inoculated with GPA-LASV as above. Beginning on day 5, 7 and 9 post-infection, favipiravir treatments were initiated in one group of animals respectively with daily s.c. injections of 300 mg/kg/d for 14 consecutive days. A control group of six guinea pigs was inoculated as above and treated with vehicle placebo beginning on day 5 post-infection. Weights and temperatures were recorded every second day. Survivors were monitored for 42 days post-infection.

### Virus detection

Tissues collected in the first efficacy experiment were tested for the presence of LASV via infectious viral titration assays as well as immunohistochemistry (IHC). For infectious titers, serial ten-fold dilutions of clarified tissue homogenates (10% weight by volume) were analyzed for viral loads on Vero cells via standard TCID_50_ methodologies. For IHC, tissue samples were inactivated and fixed with 10% formalin and processed according to standard methodologies. Thin sections were stained for viral antigen using a LASV GP2-specific monoclonal antibody (1:200 dilution, antibody L52-121-22, kindly provided by Dr. Lisa Hensley)^39^ on a Discovery XT instrument (Ventana Medical Systems, Tucson, AZ). Slides were blindly evaluated by a veterinary pathologist.

### Serology

Seroconversion to LASV infection in convalescent serum samples collected from surviving animals in the treatment studies was assessed using standard enzyme-linked immunosorbent serological assay (ELISA) methodologies with an in-house recombinant, bacterially expressed LASV nucleocapsid protein as the antigen^40^.

### Statistical analysis

Statistical differences between infectious titers in treatment groups were examined using one-way analysis of variance with Tukey-Kramer multiple-comparison posttest. Survival curves were compared using the log-rank (Mantel-Cox) test. Statistical analyses were accomplished using Prism 5 (GraphPad Software, La Jolla, CA).

## Additional Information

**How to cite this article**: Safronetz, D. *et al.* The broad-spectrum antiviral favipiravir protects guinea pigs from lethal Lassa virus infection post-disease onset. *Sci. Rep.* **5**, 14775; doi: 10.1038/srep14775 (2015).
